# Structured Additive Quantile Regression for Assessing the Determinants of Childhood Anemia in Rwanda

**DOI:** 10.3390/ijerph14060652

**Published:** 2017-06-17

**Authors:** Faustin Habyarimana, Temesgen Zewotir, Shaun Ramroop

**Affiliations:** 1 School of Mathematics, Statistics and Computer Sciences, University of KwaZulu-Natal, Pietermaritzburg Campus, Private Bag X01, Scottsville 3209, South Africa; ramroops@ukzn.ac.za; 2 School of Mathematics, Statistics and Computer Sciences, University of KwaZulu-Natal, Westville Campus, Durban 4000, South Africa; zewotir@ukzn.ac.za

**Keywords:** child malnutrition status, conditional distribution, hemoglobin, smoothing function

## Abstract

Childhood anemia is among the most significant health problems faced by public health departments in developing countries. This study aims at assessing the determinants and possible spatial effects associated with childhood anemia in Rwanda. The 2014/2015 Rwanda Demographic and Health Survey (RDHS) data was used. The analysis was done using the structured spatial additive quantile regression model. The findings of this study revealed that the child’s age; the duration of breastfeeding; gender of the child; the nutritional status of the child (whether underweight and/or wasting); whether the child had a fever; had a cough in the two weeks prior to the survey or not; whether the child received vitamin A supplementation in the six weeks before the survey or not; the household wealth index; literacy of the mother; mother’s anemia status; mother’s age at the birth are all significant factors associated with childhood anemia in Rwanda. Furthermore, significant structured spatial location effects on childhood anemia was found.

## 1. Introduction

Childhood anemia is a public health nutritional problem found mostly in developing countries. Anemia is an indicator of both poor nutrition and health [1].

It is widely known that anemia has considerable negative impacts on the health and economic welfare of the communities and the country in general. Childhood anemia can negatively affect mental development, school achievements, behavioral growth and immunity of children against disease [2,3,4,5].

According to the World Health Organisation (WHO) [4], iron deficiency anemia would be considered as a public health problem when the prevalence of hemoglobin concentration exceeds 5.0% of the population. Anemia is most prevalent among children under five years of age and in the population of pregnant women, in general. The highest prevalence of childhood anemia is in sub-Saharan countries where 67% of the childhood population are found to suffer from it and in Southeast Asia where 65.5% of the childhood population suffer from it. In Rwanda, the prevalence of childhood anemia is high but the country has made tremendous progress in reducing the prevalence of anemia among children below five years old. It has reduced the rate from 52% to 38% of children in this age group during the period from 2005 to 2010 and from 38% to 37% during the period from 2010 to 2014/2015 [6]. In other East African countries, the prevalence of childhood anemia is very high; but there is some improvement, for example, in Uganda it was 72% in 2006, 64 % in 2009 and 49% in 2011 [7]; in Tanzania in 2004–2005 it was 71.8% and 59% in 2010 [8] and in Burundi it was 45% in 2010 [9]. Crawley [5] found that 50% of all childhood anemia is caused by iron deficiency. Other causes of anemia among children in general vary according to the area of the world in which the child lives.

The childhood hemoglobin (Hb) concentration level is commonly classified based on the cut-off point [1] as follows Hb ≥ 11 g/dL as non-anemic and Hb < 11 g/dL as anemic. Therefore, in this case, the distribution is binary; or classified as Hb < 7.0 g/dL as severe anemia, 7.0 g/dL ≤Hb≤9.9 g/dL as moderate anemia, 10.0 g/dL ≤Hb≤10.9 g/dL as mild and Hb ≥11.0 g/dL as non-anemic and in this case multinomial or an ordered categorical response is considered.

Most studies in literature used a binary response variable [10,11,12,13,14] among others or a categorical ordered response variable [15,16,17]; some of them were ordinary logistic regression or spatial analysis. All these studies have considered the mean average of the response variable or Gaussian distribution but it might be possible that the upper or lower quantiles of the outcome variables depend on the covariates differently from the center. In addition, there may be a need to analyse it as a quantile of interest and this is not possible in all these models. An inference on conditional quantiles can allow the influence of explanatory variables to be assessed and give detailed information on quantiles of interests of the response variable rather than focusing solely on the mean.

Therefore, the current study aims to use structured additive quantile regression that provides the flexibility to analyse the impact of predictor variables on the different quantiles of interest of childhood hemoglobin concentration instead of the mean distribution. In addition, this model allows the possible nonlinearity effects of continuous covariates to be accounted for, the possible structured spatial effects on childhood anemia to be captured and possible heterogeneity among the variables to be catered for. In the current scientific setting, moderate and mild childhood anemia are our main interest. The researchers have chosen moderate and mild childhood anemia because of their high prevalence rate compared to severe anemia 1%. Therefore, the quantiles of interest were fixed at *θ* = 0.15, 0.21 and these quantiles correspond empirically to 15% and 21%—the prevalence of moderate and mild childhood anemia in Rwanda, respectively [6]. If, for example, the current interest was any type of childhood mild anemia, then the quantile of interest should be fixed accordingly at *θ* = 0.37 which corresponds to a 37% prevalence of childhood anemia in Rwanda [6]. It was hoped that the findings of this study will help the policy makers and other public health related institutions to properly understand the determinants of childhood anemia in Rwanda and visualize the spatial components of childhood anemia at a district level. Hence, this can help them to improve the current strategies to reduce its prevalence.

## 2. Materials and Methods

### 2.1. Data Source

This study utilises data from the 2014/2015 RDHS data set. The survey was conducted from 9 November 2014 to 8 April 2015; 12,793 households were selected. The survey used the sampling frame from the 2012 population and housing census. The two-stage clustering sampling technique with stratification was used to collect the data. During the first stage, 113 and 379 clusters were obtained from urban and rural areas respectively, and in the second stage, systematic sampling was used among the selected households. More details on sampling techniques and data collection can be found in [6]. This survey used three questionnaires: a questionnaire for men, a questionnaire for households and a questionnaire for women. The household questionnaire collected information on dwelling characteristics, hemoglobin measurements of women and children, height and weight of women aged 15–49, men aged 15–59 and children aged 0–5 years among others. The questionnaire for women provided information on maternal and child health among others. The RDHS 2014/2015 provided a children’s dataset among others and this was used in this study. RDHS 2014/2015 selected 13,497 women aged 15–49 and 7856 children under five years old and testing for malaria and anemia was only done in a 50% subsample of households for women and children.

#### 2.1.1. Dependent Variable

The outcome variable of interest is the childhood hemoglobin concentration in blood measured in grams per deciliter (g/dL). RDHS 2014/2015 provided both unadjusted and adjusted childhood hemoglobin concentration level to altitude and the latter was used in this study.

#### 2.1.2. Independent Variables

The set of explanatory variables used in this study was also used in various studies modeling childhood anemia; [10,11,12,13,15,16,17,18,19] just to name few. Consequently, this forms the theoretical framework that will underpin the current research. These variables are classified as socio-economic, demographic and geographic variables: sex of the child; child’s malnutrition status (underweight or not, stunted or not and wasted or not); whether the child had fever, cough or diarrhea in two weeks prior to the survey or not; the birth order of the child; the child’s birth weight; whether the child had received the drug for intestinal worms or a vitamin A supplementation in the six months prior to the survey; whether the child slept in a bed with a mosquito net; whether the child had eaten meat, fish or poultry in the six weeks before the survey; the mother’s anemia status; the mother’s education level; the mother’s age at the birth (continuous); the place of residence; the wealth index of the family of the child (grouped as poor, middle and rich); the mother’s literacy; mother’s level of education; the duration of breastfeeding (continuous); the child’s age in months (continuous); the gender of the household head; and the district the child lived in, but this was used as a spatial variable where all 30 districts were labeled exactly as the label on the map.

### 2.2. Statistical Analysis

The quantile regression model allows for the analysis of the effect of covariates on the entire conditional distribution of the response variable, instead of the conditional mean of the response variable [20,21]. Given a fixed quantile θ∈(0, 1), therefore, the general additive conditional quantile model corresponding to continuous response $Y_{i}$ is given by

(1) $$Q_{Y_{i}}\left(\theta |x_{i},u_{i}\right)=n_{\theta i}=x_{i}^{\prime }\beta_{\theta }+\overset{n}{\underset{k=1}{\sum }}f_{\theta k}\left(u_{ki}\right)+b_{\theta i}$$

where $n_{\theta i}$ is the conditional *θ*th quantile outcome given $x_{i},u_{i}$; $x_{i}$ is the vector of m categorical covariates, $u_{i}$ is the vector of n metric or continuous covariates; $\beta_{\theta }$ is the vector of fixed coefficients of categorical variables and finally $f_{\theta k}$ is a vector of smoothing functions (which may be continuous or spatial or both) [22,23] and $b_{\theta i}$ is unstructured random effect. Alternatively, Equation (1) can be represented in the form of a minimization problem as:

(2) $$arg\;\underset{n_{\theta }}{\min}\sum_{i=1}^{n}\rho_{\theta }\left(y_{i}-n_{\theta i}\right),\;\mathrm{with}\;\rho_{\theta }\left(u\right)=\{\begin{array}{c} u\theta ,\;u\geq 0\\ u\left(\theta -1\right)\;u<0 \end{array}$$

Equation (2) is known as a check function or loss function [21] and $n_{\theta }$ is the same as in Equation (1). In the case of simple linear quantile regression, Equation (2) can be solved using a linearization problem [22,24] among others, but in the case of the non-linear case such as the structured additive quantile model, the estimation requires the Bayesian inferential approach. The method assumes that $Y_{i}$ has an asymmetric Laplace distribution; see [24,25,26] among others, for more details.

In the Bayesian analysis framework, the unknown functions for the metric, the spatial effects, regression coefficients for categorical covariates and all variance parameters have to be assigned suitable prior distributions. Within the ambit of this study, diffuse priors are considered for the fixed effects; the continuous covariates are modeled by assuming seconder order random walk priors [27]. The structured spatial effect was modeled through Gaussian Markov Random Fields (GMRF) specified as an intrinsic conditional autoregressive model [28]. The intrinsic conditional autoregressive models have flexible neighborhood structures that are very useful in the analysis of the data used in this study.

The common and well known method to estimate the Bayesian posterior marginal distribution is the Markov Chain and Monte Carlo (MCMC). Recently, the authors of [29,30] introduced integrated nested Laplace Approximation (INLA) and this method was found to be an alternative method to MCMC. In this research, the INLA was used because its solution converges faster than MCMC in the case of quantile regression models and many other cases [29,31].

### 2.3. Model Selection

In order to identify the factors associated with childhood anemia, this study fitted the following models.

$$\mathrm{Model}\;1:\;Y_{i}=x_{i}^{\prime }\beta_{i}+\varepsilon_{i}$$

Model 1 is known as the simple linear regression model, where $x_{i}$ stands for any predictor or independent variable considered in the model and $\beta_{i}$ is its coefficient and $\varepsilon_{i}$ is the error terms in the model.

$$\mathrm{Model}\;2:\;n_{\tau i}=x_{i}^{\prime }\beta_{\theta }$$

where$\;x_{i}$ stands for all categorical variables and their effect is summarized in $\beta_{\theta }$; this model is known as linear quantile regression

$$\mathrm{Model}\;3:n_{\theta i}=x_{i}^{\prime }\beta_{\theta }+f_{\theta 1}\left(child\;age_{i}\right)+f_{\theta 2}\left(Mothe\;age_{i}\right)+f_{\theta 3}\left(Duration\;of\;breastfeeding_{i}\right)$$

where Model 3 combines the fixed effect and nonlinear effects, $x_{i}$ and $\beta_{\theta }$ have the same meaning as in Model 2; Model 3 is known as additive quantile regression.

$$\mathrm{Model}\;4:\;n_{\theta i}=x_{i}^{\prime }\beta_{\theta }+f_{\theta }\left(district_{d}\right),\;d=1,2,\ldots ,30$$

Model 4 combines the fixed effects and spatial effects, where district is considered as spatial and this model is known as spatial quantile regression

$$\mathrm{Model}\;5:\;n_{\theta i}=x_{i}^{\prime }\beta_{\theta }+f_{\theta 1}\left(child\;age_{i}\right)+f_{\theta 2}\left(Mothe\;age_{i2}\right)+\;f_{\theta 3}\left(Duration\;of\;breastfeeding_{i3}\right)+f_{d\theta }\left(district_{d}\right),\;d=1,2,\;\ldots ,30$$

Model 5 extends Model 3 to include structured spatial effects and therefore, Model 5 is known as a structured spatial additive quantile regression.

## 3. Results

In order to make sure that the estimates obtained from the current study were represented at national level, the survey weights provided in Rwanda Demographic and Health Survey data set were used in the analysis. The frequencies and cross tabulation analysis were done using the SPSS Complex survey commands (available in SPSS version 23) correcting for weighting and stratification of random samples, for each outcome-predictor relationship. The Chi-square values were used to assess the association between the outcome variables and the predictor variables.

### 3.1. Exploratory Data Analysis

The results of cross-tabulation analysis are summarized in Table 1. The childhood hemoglobin concentration was categorized as severe, moderate, mild and non-anemic. Based on cross-tabulation analysis, it was found that the presence of fever or cough; the incidence of diarrhea; underweight; wasting; stunting; vitamin A supplementation; drug for intestinal worms; mother’s anemia level; mother’s level of education; mother’s literacy; place of residence; and wealth index of the family are associated with childhood anemia at a 5% level of significance. The prevalence of anemia is higher in male children than in female children. From Table 1, it is observed that 36.9% of male children are anemic, and 36.4% of female children are anemic. It is also observed from the same table that a child who had a fever in the two weeks prior to the survey had a higher prevalence of childhood anemia than a child who did not have a fever in the two weeks before the survey.

It is observed from Table 1 that the prevalence of anemia is higher among children who did not receive vitamin A supplementation in the six weeks prior to the survey. The rate is 45.9% against 35.5% among children who had received the vitamin A supplementation (*p*-value < 0.001).

It was also observed that a child’s nutritional status is highly associated with childhood anemia (underweight, *p* < 0.001; stunting, *p*-value = 0.002 and wasting, *p*-value < 0.001). A significant statistical association between childhood anemia and drugs taken for intestinal worms was noted from the results (*p*-value < 0.001). Childhood anemia is higher among children who did not receive the drug for intestinal worms, than children who had received them. It was also found that there is a significant association between childhood anemia and the mother’s anemia status (*p*-value < 0.001). The prevalence of anemia was higher at 47.8% among children born to anemic mothers than those children born to non-anemic mothers where the rate was 34.2%. The literacy of the mother is highly associated with childhood anemia (*p*-value < 0.001). Anemia is higher among children born to an illiterate mother and this is about 40.6% of those children. In the case of children born to a literate mother, the rate was only 35.5% of the population. In order to account for possible multicollinearity and confounding among the covariates, any variable with p-value less than 0.2 was included in the structured additive quantile regression model. A two-way interaction effect between the explanatory variables was included in the analyses but none was found to be statistically significant and it was dropped from the model.

**Figure 1:**
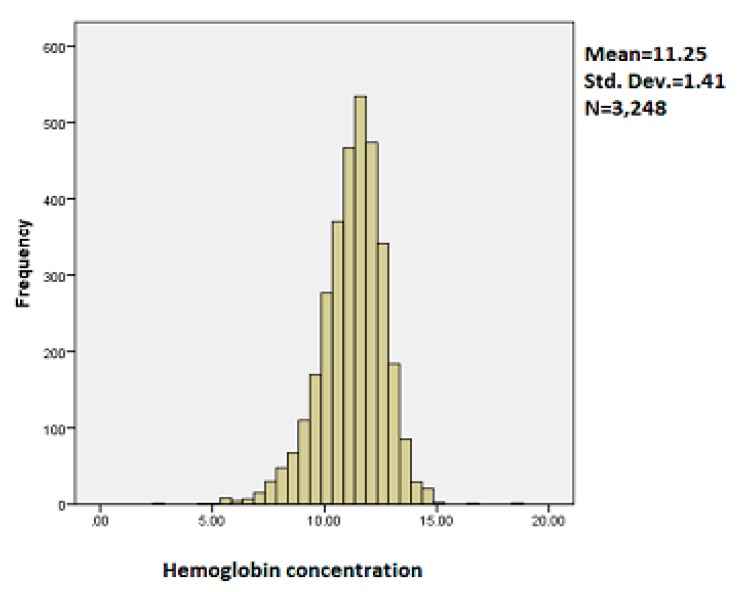
Histogram of childhood hemoglobin concentration.

The histogram of the outcome variable is presented in the Figure 1.

It is observed from the histogram of childhood hemoglobin concentration that the mean is 11.25, standard deviation was 1.41 and the number of children was 3248. It is observed from Figure 1 that the distribution of childhood hemoglobin concentration is slightly skewed to the left.

### 3.2. Results of Structured Additive Quantile Regression Analysis

In Model 1, known as the simple linear regression model, we fit all predictor variables as fixed effects. In Model 2, known as linear quantile regression, we fit all predictor variables as fixed effect. In Model 3, we fit categorical variables as fixed effects and continuous variables as non-linear effect. In Model 4, we combined fixed effects and structured spatial effects and finally in Model 5, we considered fixed effects, non-linear effects and structured spatial effects.

#### 3.2.1. Model Fit

Deviance Information Criteria (DIC) were used to select the best model. The smaller the DIC, the better the model fit [32]. The summary of DIC for all considered models is reported in Table 2. It is observed from the table that Model 5 has smaller DIC compared to Models 1–4. Therefore, Model 5 is considered in the final analysis of this study.

#### 3.2.2. Effects of Categorical Variables on Childhood Anemia

In the interpretation that follows, any categorical variable that is positively associated with childhood hemoglobin concentration reduces childhood anemia (moderate 15%, mild 21% and any type of anemia 37%). Conversely, any categorical variable that is negatively associated with childhood hemoglobin concentration increases the incidence of childhood anemia.

The results of the fixed effects are summarised in Table 3. It can be observed from the table that the wealth index of the family has a significant effect on adjusted childhood hemoglobin concentration. In general, the adjusted childhood hemoglobin concentration increases with increasing the wealth index of the family. In addition, the coefficient (effect) increases with increasing the quantile.

It can be observed from the table that a child born to a rich family is less likely to be anemic compared to a child born to a poor family and the effect increases with increasing the quantile. This may be due to the fact that a rich family has enough income to contribute to the household welfare.

Child nutrition status (wasting or being underweight) has a significant effect on adjusted childhood hemoglobin concentration. It can be observed from the results that malnutrition has a negative effect on childhood hemoglobin concentration. This implies that anemia is therefore more attributable to underweight children and/or children that suffer from wasting.

Vitamin A supplementation has been seen from the results to have a positive significant effect on adjusted childhood hemoglobin concentration in all quantiles. It can be observed from Table 3 that a child who had received vitamin A supplementation in the six weeks prior to the survey is less likely to be anemic compared to a child who did not receive a vitamin A supplementation, in the six weeks before the survey.

The gender of the child, according to the results, significantly affects the incidence of childhood anemia. It is noted from the results that being a male child positively affects the childhood hemoglobin concentration compared to being a female child. This means that childhood anemia is more attributable to a female child than a male child.

Fever was found to be a significant predictor of childhood anemia. The results revealed that a recent incident of fever has a significant negative effect on adjusted childhood hemoglobin concentration in all quantiles. A child who had a fever in the two weeks prior to the survey, showed negative effects on adjusted childhood hemoglobin concentration across all quantiles where the negative effect decreases with increasing the quantile. It can be deduced therefore that a child who had fever in the two weeks before the survey is more likely to be anemic than a child who did not have a fever in the two weeks prior to the survey.

The results of Table 3 show that a cough has a significant negative effect on childhood hemoglobin concentration and this effect decreases with increasing the quantile. It was noted from the results that a child who had a cough in the two weeks prior to the survey was more likely to be anemic than a child who did not have a cough in the two weeks prior to the survey.

Literacy levels also significantly affect the incidence of childhood anemia. It was noted from the results that childhood hemoglobin concentration in blood was negatively affected among children born to an illiterate mother compared to the hemoglobin concentration of children that are born to a literate mother.

The mother’s anemia status has a significant effect on her child’s anemia status. It was observed from the results in Table 3 that a child born to an anemic mother is more likely to be anemic than a child born to a non-anemic mother.

The mother’s body mass index has a significant effect on the adjusted hemoglobin concentration in the mean model. It was noted from Table 3 that a child born to an underweight mother is more likely to be anemic as compared to a child born to a normal or obese mother (BMI ≥ 18.5).

#### 3.2.3. Nonlinear Effects on Childhood Anemia

**Figure 2:**
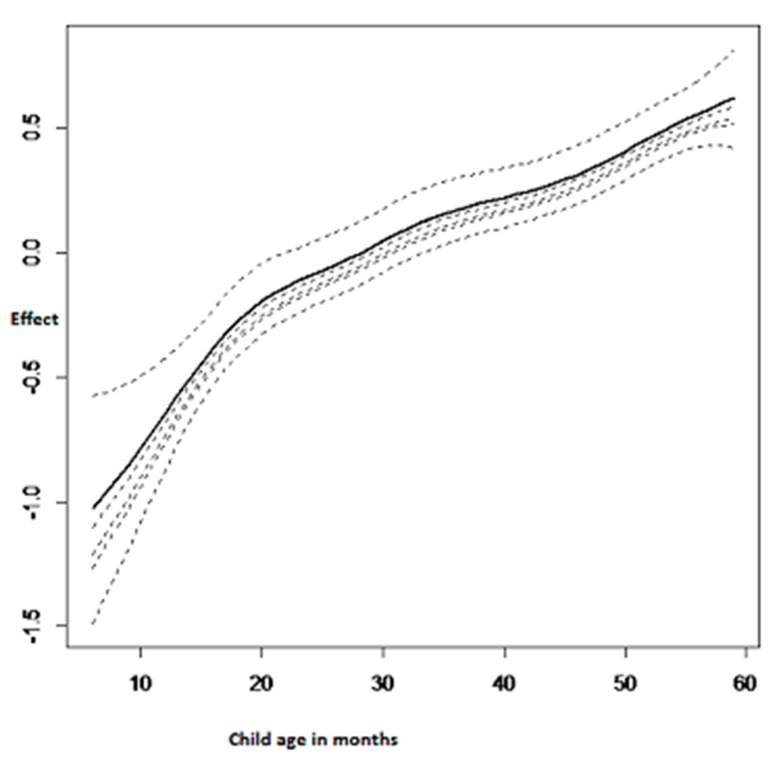
Nonlinear effects of the child’s age on adjusted childhood hemoglobin concentration.

Figure 2 summarises the nonlinear effects of the child’s age on adjusted childhood hemoglobin concentration. It was observed that the child’s age had a significant positive effect on adjusted childhood hemoglobin concentration. This means that the child’s age has a negative effect on childhood anemia. Therefore, childhood anemia decreases with the increasing age of the child.

**Figure 3:**
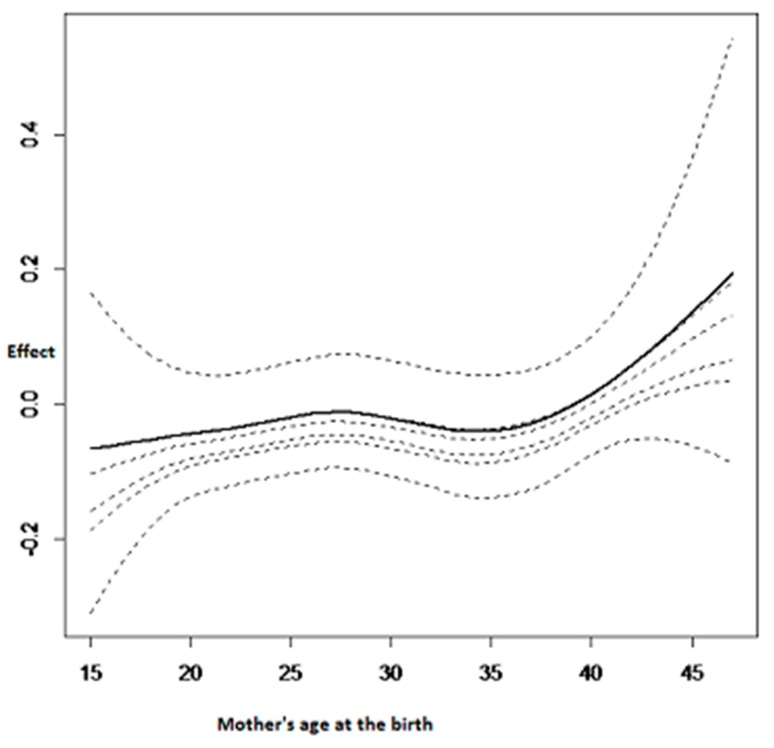
Nonlinear effects on childhood hemoglobin concentration: mother’s age at the birth.

The nonlinear effect of the mother’s age at birth is summarised in Figure 3. The age of the mother at the birth is also very important for determining childhood anemia status. To give birth at an earlier age (before 20 years old) has a negative effect on childhood hemoglobin concentration and as a consequence it increases the chances of the child being anemic. In contrast, a positive effect was observed when the mother’s age at birth was between 20 and 30 years old. Thereafter, a U shape was noted with a minimum at almost 35 years old and then the childhood hemoglobin concentration increases afterward, as can be seen in Figure 3.

**Figure 4:**
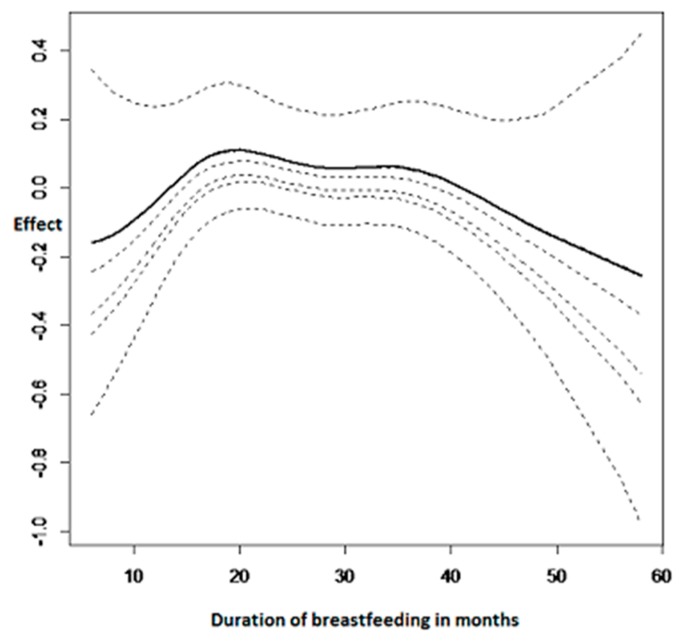
Nonlinear effects on childhood hemoglobin concentration: duration of breastfeeding.

Figure 4 summarises the nonlinear effects of duration of breastfeeding on adjusted childhood hemoglobin concentration. It can be observed from the results that breastfeeding was also a very important factor to consider when determining childhood anemia status.

Breastfeeding a child for less than 10 months shows a decrease in childhood hemoglobin concentration and this means that it increases the risk of childhood anemia. Afterwards, it is an inverse U shape where the maximum is breast feeding for a period of 25 months.

#### 3.2.4. Spatial Effects

**Figure 5:**
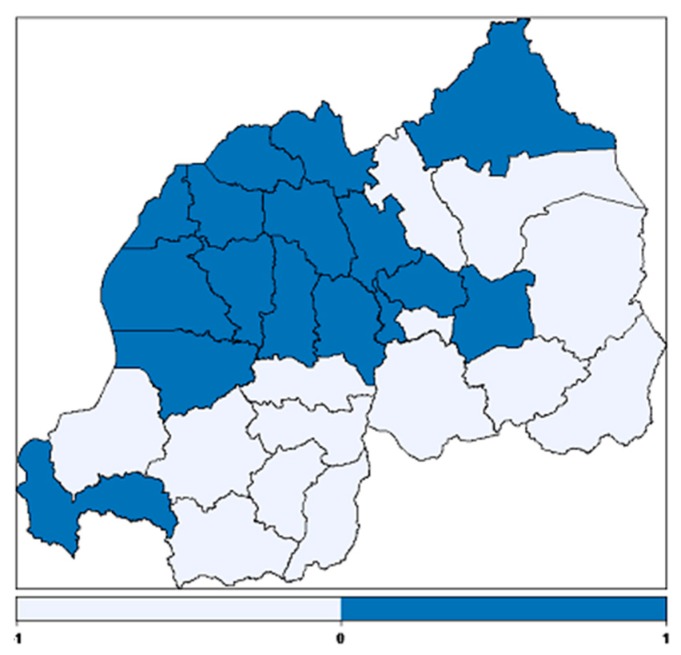
Structured spatial effects on childhood hemoglobin concentration where dark blue colour regions depicts lower risk and light blue depicts higher risk of anemia.

The structured spatial effect on adjusted childhood hemoglobin concentration is presented in Figure 5. The study found significant spatial effects on childhood anemia. The districts in dark blue have a positive effect on childhood hemoglobin concentration and therefore they are at lower risk for the incidence of childhood anemia and the districts in light blue have a negative effect on childhood hemoglobin concentration and therefore they are at higher risk for the incidence of childhood anemia.

## 4. Discussion

The quantile regression has an advantage over binary logistic regression in that it allows the impact of predictor variables on the complete distribution of the response variable to be determined. In addition, outliers and extreme data are usually less influential in quantile regression due to the inherent robustness of quantile regression.

This study used a structured spatial additive quantile regression model to identify the determinants of childhood anemia and to map the spatial variability of childhood anemia.

This approach is very powerful. In addition to the advantages of quantile linear regression over the mean models, it also allows the possible non-linearity effect of continuous covariates and the possible structured additive spatial effects on childhood hemoglobin concentration to be accounted for.

The inference in this study was fully Bayesian. It is well known that the inference in a Bayesian framework is done based on MCMC. However, recently, it was shown that INLA can produce similar results to MCMC in a shorter time and sometimes more accurately [29,31]. Therefore, the R-INLA 3.3.1 package was used to estimate the posterior marginal distributions.

In general, the results found in this study agreed with major findings from other related studies.

It was observed from the results that fever has a significant negative effect on childhood hemoglobin concentration in all quantiles of interest and therefore a child having a fever increases the likelihood of childhood anemia. This is consistent with the results found by Gayawan et al. [13] and many others. This may be due to the fact that fever is mostly accompanied by other morbidity such as diarrhea, malaria, cough and these are also known to positively affect childhood anemia.

This study revealed that children born to literate mothers are less likely to be anemic compared to children born to illiterate mothers. In addition, the negative effect was higher in lower quantiles than higher quantiles. This might be due to the fact that a literate mother has better information on nutritional education and adequate healthcare that may help to protect the child against anemia [18,33]. In addition, a literate mother may generate more income easily than an illiterate mother and all these contribute to the household well-being. This finding is consistent with that of [16].

The findings from this study showed that childhood anemia is less common in male children compared to female children. These findings are in line with [34]. In contrast, a study done in Kenya by Ngesa et al. [35] found that anemia was higher among male children than female children.

It was also found that vitamin A supplementation increases the childhood hemoglobin concentration and thereby decreases childhood anemia. This finding is in agreement with [36].

The results show that the nutritional status of the child has a significant effect on childhood anemia. An underweight child or a child suffering from wasting was found to be more anemic than a non-underweight child or a child that does not suffer from wasting. These findings are consistent with that of [15,37]. As underweight and wasting are long-term and short-term indicators of malnutrition respectively, the consequences of the result were that a malnourished child experienced higher risk of developing anemia than a nourished child. Since anemia and malnutrition often share common causes, it is expected that multiple nutrition problems would occur in the same individual [19,37,38].

The findings further show that children born to an anemic mother were more likely to be anemic than children born to a non-anemic mother. This may be explained by the fact that maternal anemia during pregnancy may contribute, for example, to low birth weight and this was found to positively affect the incidence of childhood anemia; as was found by Cessie et al. [39]. These findings are in agreement with that of [37,40] among others.

The findings showed a significant effect of household wealth quantile on the childhood hemoglobin concentration. The children born to a rich family have been found to have a reduced risk of childhood anemia compared to those children that are born to poor families and the effect was higher in upper quantiles. This might be due to the fact that the rich families can afford to buy nutritious food which may help to prevent anemia. These findings are similar to that of [12,13,19,35]. However, the study did not find a significant difference between a child born in a middle-income family and one born in a poor family and the association with childhood hemoglobin concentration.

The mother’s body mass index was also found to significantly affect the childhood hemoglobin concentration. The results showed that children born to an underweight mother were more likely to be anemic compared to children born to a mother who was not. These findings are consistent with that of [19].

The study did not find any statistically significant association between the place of residence, drug intake to eradicate intestinal worms, diarrhea, the gender of the household head, the use of a mosquito bed net, the stunting of childhood growth and childhood hemoglobin concentration.

The study found significant non-linear effects between the duration of breastfeeding, the child’s age, and the mother’s age at the birth of the child and childhood hemoglobin concentration. It was found that childhood anemia decreases with increasing the age of the child. These results are in agreement with [12] among others. This means that older children have less chance of getting anemia, than younger children. The findings also show that younger mothers were more likely to have anemic children. This may be due to the fact that younger mothers require more iron for their own growth and this affects the childhood hemoglobin concentration. This result is in agreement with previous findings such as [12] among others.

The duration of breastfeeding was also found to significantly affect the incidence of childhood anemia. A child who had been breastfed for less than ten months showed a decrease in childhood hemoglobin concentration. In contrast, childhood hemoglobin concentration is found to be at an increased level if they had been breastfed for between ten months and thirty months. The recent study on exclusive breastfeeding by Wang et al. [41] found that increasing exclusive breastfeeding was associated with childhood anemia.

The spatial variation of childhood anemia was examined. On the map, the dark blue colour corresponds to the regions where there is a positive effect on childhood hemoglobin concentration and thus a low risk of childhood anemia. The light blue colour corresponds to the regions where there is a negative effect on childhood hemoglobin and thus a high risk of childhood anemia. It can be seen from the map that most regions at higher risk are in the eastern province and southern province; this finding is in line with [6]. The eastern province is characterised by lowland areas that have higher temperatures and therefore are an endemic region of malaria. The evidence for the development of anemia among young children living in malaria endemic regions has been reported elsewhere [14,42]. The regions at lower risk of childhood anemia are mostly in the northern province, Kigali city, followed by the western province. This is not surprising, because the northern province is mostly a highland region where the malaria epidemic is very low. The higher prevalence of childhood anemia in some districts of the highland region may be due to poverty of the household members or the poor nutritional status of the children.

## 5. Study Limitations

The current study used cross-sectional data and this data cannot determine the causality, therefore a longitudinal study is suggested to address this problem.

## 6. Recommendation

The findings of the study suggest improving vitamin A supplementation among children under five years of age. They also suggest an eradication of illiteracy as well as poverty among households and the eradication or prevention of fever and cough among children.

## 7. Conclusions

This study has revealed that having a fever or a cough; the mother’s illiteracy; having an anemic mother; the child’s nutritional status (including being underweight and suffering from wasting) all have significant negative effects on adjusted childhood hemoglobin concentration and thereby increase the incidence of childhood anemia. In contrast, the study revealed that the greater the household wealth, the lower the childhood anemia. It was also found that being a male child and receiving vitamin A supplementation in the six weeks prior to the survey has a positive effect on adjusted childhood hemoglobin concentration and thereby reduces the risk of having childhood anemia. Furthermore, based on continuous covariates, the study found a significant non-linear effect of the child’s age, duration of breastfeeding and the mother’s age at the birth on childhood hemoglobin concentration. It was found that childhood anemia decreases with increasing the age of the child. Significant spatial structured additive effects on childhood anemia were also found.

The significant variables in our model discussed in the fixed effects can help the policy makers and public health related institutions to design programmes for interventions. The identification of higher prevalence districts from the model generated map can help to provide insights to design campaign programmes, undertake adequate intervention to those districts and channel resources efficiently.

## Figures and Tables

**Table 1:** Childhood anemia by categorical variables.

Variable	Category	Anemic (%)	Not Anemic (%)	Pearson Chi-Square *p*-Value
Sex of the child	male	607 (36.9%)	1038 (63.1%)	0.125
	female	583 (36.4%)	1020 (63.6%)	
Birth order	1st	330 (35.6%)	830 (64.4%)	0.374
	2–3	458 (35.7%)	595 (64.3%)	
	4–5	230 (38.9%)	273 (61.1%)	
	6+	172 (38.7%)	361 (61.3%)	
Did child eat meat, fish or poultry	yes	35 (53.0%)	31 (47.0%)	0.097
	no	736 (42.7%)	987 (57.3%)	
Had fever in the last two weeks	yes	298 (44.4%)	373 (55.6%)	<0.001
	no	892 (34.6%)	1685 (65.4%)	
Had coughing in the last two weeks	yes	372(39.0%)	581(61.0%)	0.068
	no	818 (35.6%)	1477 (64.4%)	
Had diarrhea in the last two weeks	no	993 (35.4%)	1816 (64.6%)	<0.001
	yes	197 (44.9%)	242 (55.1%)	
Stunted	no	740(34.8%)	1388 (65.2%)	0.002
	yes	451 (40.2%)	670 (59.8%)	
underweight	no	1157 (36.3%)	2034 (63.7%)	<0.001
	yes	34 (58.6%)	24 (41.4%)	
Wasting	no	992(35.1%)	1838 (64.9%)	<0.001
	yes	199 (47.5%)	220 (52.5%)	
Child’s birth weight in kg	Low (<2500 g)	57 (38.3%)	92(61.7%)	0.643
	Higher (≥2500 g)	1039 (36.5%)	1817 (63.65)	
Received Vitamin A	yes	1005(35.5%)	1840 (64.7%)	<0.001
	no	185 (45.9%)	218 (54.1%)	
Had received drugs for intestinal worms	yes	873 (33.1%)	1766 (66.9%)	<0.001
	no	317 (52.1%)	292 (47.9%)	
Mothers’ education level	No education	189 (39.5%)	289 (60.5%)	0.153
	Primary	857 (36.0%)	1523 (64.0%)	
	Secondary a	126(38.8%)	199 (61.2%)	
	higher	18(27.3%)	48 (72.7%)	
Mother’s anemia level	anemic	279 (47.8%)	305 (52.2%)	<0.001
	no	912 (34.2%)	1753 (65.8%)	
Mother’s literacy	Yes	877 (35.5%)	1594(64.5%)	0.016
	No	313 (40.6%)	464(59.7%)	
Household size	1–3	203 (38.6%)	323 (61.4%)	0.309
	4 and more	987 (36.3%)	1735 (63.7%)	
Place of residence	Urban	156 (30.0%)	364 (70.0%)	<0.001
	rural	1034 (37.9%)	1694 (62.1%)	
Wealth index	Poor	617 (40.4%)	910 (59.6%)	<0.001
	Middle	240 (37.0%)	408 (63.0%)	
	Rich	333 (31.0%)	740 (69.0%)	
Mother BMI	Less 18.5	58 (40.6%)	85 (59.4%)	0.183
	≥18.5	1133 (36.5%)	1973 (63.5%)	
Slept with a mosquito-net	Yes	1007 (36.3%)	1770 (63.7%)	0.280
	no	183 (38.9%)	288 (61.1%)	
Household head	female	235 (38.5%)	375 (61.5%)	0.288
	male	956 (36.2%)	1683 (63.8%)	

**Table 2:** Model comparison based on Deviance Information Criteria (DIC).

Statistics	Model 1	Model 2	Model 3	Model 4	Model 5
DIC	10,757.44	10,750.7	10,707.7	10,996.9	10,629.84
pD	15.90	16.005	23.74	32.98	43.82
D	10,725.64	10,718.7	10,660.2	10,931	10,542.2

**Table 3:** Summary of the fixed effects of childhood anemia.

Variable	Posterior Mean	Standard Deviation	0.025	0.15	0.21	0.37	0.50	0.975
Intercept	11.1994	0.2253	10.7571	10.9657	11.0176	11.1247	11.1994	11.6414
Sex of the child (female = ref) Male	0.1194	0.0452	0.0307	0.0725	0.0829	0.1044	0.1194	0.2080
Had fever (no = ref) Yes	−0.3511	0.0649	−0.4785	−0.4184	−0.4035	−0.3727	−0.3511	−0.2239
Had cough (no = ref) Yes	−0.1359	0.0572	−0.2483	−0.1953	−0.1821	−0.1549	−0.1359	−0.0236
Wasting (no = ref) Yes	−0.3214	0.0715	−0.4618	−0.3956	−0.3791	−0.3451	−0.3214	−0.1811
Underweight (no = ref) Yes	−0.2421	0.1773	−0.5902	−0.4260	−0.3851	−0.3009	−0.2421	0.1057
Mother’s anemia (Yes = ref) No anemic	0.4427	0.0600	0.3248	0.3804	0.3943	0.4228	0.4427	0.5605
Mother’s literacy (yes = ref) No	−0.1736	0.0562	−0.2839	−0.2319	−0.2189	−0.1922	−0.1736	−0.0634
Mother’s BMI (≥18.5 = ref) <18.5	−0.0857	0.1115	−0.3046	−0.2014	−0.1757	−0.1227	−0.0858	0.1329
Wealth index (poor = ref) Middle	0.0113	0.0624	−0.1113	−0.0535	−0.0391	−0.0094	0.0113	0.1337
Rich	0.2259	0.0568	0.1143	0.1669	0.1800	0.2070	0.2259	0.3374
Received vitamin (no = ref) Yes	0.1441	0.0732	0.0005	0.0682	0.0851	0.1199	0.1441	0.2877
